# Progressive muscle relaxation alleviates anxiety and improves sleep quality among healthcare practitioners in a mobile cabin hospital: a pre-post comparative study in China

**DOI:** 10.3389/fpsyg.2024.1337318

**Published:** 2024-04-30

**Authors:** Yuding Luo, Juan Du, Junqiu Wang, Pingchuan Liu, Zhongli Shi, Yu He, Guangyao Che, Ke Huang, Jian Wang

**Affiliations:** ^1^Department of Neurology, The Affiliated Hospital, Southwest Medical University, Luzhou, China; ^2^Department of Neurology, Ya'an People's Hospital, Ya'an, China; ^3^Nursing Department, Ya'an People's Hospital, Ya'an, China; ^4^Department of Orthopedics, The Affiliated Traditional Chinese Medicine Hospital, Southwest Medical University, Luzhou, China; ^5^Department of Gastroenterology, Ya'an People's Hospital, Ya'an, China; ^6^Department of Respiratory and Critical Care Medicine, Ya'an People's Hospital, Ya'an, China; ^7^Medical Department, Ya'an People's Hospital, Ya'an, China

**Keywords:** progressive muscle relaxation, COVID-19, sleep quality, anxiety, mobile cabin hospital, healthcare practitioners

## Abstract

**Objective:**

To investigate the anxiety levels, sleep quality and potential risk factors of healthcare practitioners involved in the management of COVID-19 patients in a mobile cabin hospital, and further to assess the impact of progressive muscle relaxation (PMR) on their anxiety levels and sleep quality.

**Methods:**

We conducted a pre-post self-controlled trial. Healthcare practitioners meeting the inclusion criteria underwent daily 30-min PMR sessions for seven consecutive days. The Pittsburgh Sleep Quality Index (PSQI) and Hamilton Anxiety Scale (HAMA) were used to assess the anxiety and sleep quality of subjects pre- and post-intervention. Statistical analysis was performed using the Wilcoxon test, Mann–Whitney U test, Kruskal-Wallis H test, and Spearman rank correlation.

**Results:**

A total of 94 participants completed the study. No statistically significant differences in HAMA or PSQI total scores were observed between groups categorized based on demographic variables such as age, sex, and years of education (*p* > 0.05). The PSQI total score and its components (excluding sleep medication usage) exhibited a positive correlation with the HAMA total score and its psychological anxiety component (*p* < 0.05), and a correlation was observed between somatic anxiety manifestations and several components of the PSQI. The PSQI total scores before and after intervention were 10.0 (8.0, 13.0) and 8.0 (6.0, 9.0) respectively (*p* < 0.001); the HAMA total scores were 8.0 (5.0, 13.0) and 6.0 (4.0, 9.5) respectively (*p* < 0.001). The detection rates of poor sleep and anxiety states, along with their severity, significantly decreased post-intervention (*p* < 0.001).

**Conclusion:**

Healthcare practitioners experience prominent anxiety and sleep issues in the mobile cabin hospital. PMR can be an effective intervention for improving the anxiety and sleep quality of healthcare professionals during support periods in the mobile cabin hospital. However, trials with larger samples are necessitated to further affirm these preliminary findings.

## 1 Introduction

The worldwide outbreak of the COVID-19 pandemic has presented unparalleled challenges to the field of medical and nursing practices. Mobile cabin hospitals, alternatively refer to as Fangcang shelter hospitals, modular hospitals, or field hospitals, assume a pivotal role in addressing extensive epidemics and delivering essential medical services to a substantial population of COVID-19 patients (Chen et al., 2020; Li et al., 2022). However, mobile cabin hospitals frequently encounter formidable challenges, including a scarcity of medical resources, demanding work schedules, and secluded environments, which not only evoke apprehension among medical personnel regarding their health risks but also significantly impact their mental state and behavior. A national survey conducted among Italian physicians during the COVID-19 pandemic revealed that healthcare practitioners experienced a range of psychological issues, including anxiety, depression, attention deficits, concentration difficulties, anorexia, mood swings, apathy, and feelings of frustration. Insomnia emerged as one of the most prevalent symptoms, with 58.3% of participants reporting experiencing sleep disturbances or difficulties, while anxiety symptoms were reported by 24.6% of respondents (Romiti et al., 2023). A meta-analysis revealed that among frontline healthcare workers involved in managing COVID-19, the prevalence rates of anxiety, depression, and insomnia were 37, 36, and 32%, respectively (Sun et al., 2021). This evidence delineates a pronounced manifestation of psychological distress among frontline healthcare workers amidst the COVID-19 pandemic. Such stressors are poised to amplify both physical exhaustion and emotional strain encountered by medical personnel, thereby posing a plausible threat to their cognitive faculties, including concentration and decision-making aptitudes. As a corollary, this could culminate in a decrement of overall work efficacy and performance (Shiri et al., 2022). Hence, it is urgently needed to identify a straightforward and effective approach to mitigate anxiety and sleep-related challenges among healthcare practitioners in mobile cabin hospitals.

Progressive muscle relaxation (PMR) is a non-invasive, economical, effective, and easy-to-implement autogenic training technique and relaxation method initially developed by Edmund Jacobson in 1938. This technique entails the systematic contraction and subsequent relaxation of distinct muscle groups coupled with deep breathing until comprehensive relaxation of the entire body is attained (Alhawatmeh et al., 2022). PMR can enhance both physical and mental relaxation by diminishing sympathetic nervous system activity, thereby reducing anxiety and stress levels and improving sleep quality (Ferendiuk et al., 2019). PMR were found to be effective in improving sleep quality and reduce anxiety levels in patients with fractures, chronic obstructive pulmonary disease, malignancy, and chronic pain (Xie et al., 2016; Qaseem et al., 2017; Seyedi Chegeni et al., 2018; Wang et al., 2024). In patients with COVID-19, PMR has also demonstrated benefits in ameliorating sleep quality, anxiety, depression, disease severity, and overall quality of life (Seid et al., 2023). Liu et al. investigated the effects of PMR administered for 30 min per day for 5 consecutive days on COVID-19 patients in isolation wards, which showed a significant reduction in anxiety levels and improvement in sleep quality in the intervention group (*n* = 25) compared to the control group (*n* = 26) post-intervention (Liu et al., 2020). Similar results were observed in two other clinical studies, despite variations in the PMR strategies employed (Xiao et al., 2020; Özlü et al., 2021). However, the effect of PMR on anxiety and sleep quality among medical professionals in mobile cabin hospitals has been scarcely reported.

The aim of this study was to investigate the anxiety levels, sleep quality and potential risk factors of healthcare practitioners involved in the management of COVID-19 patients in a mobile cabin hospital, and further to assess the impact of PMR on their anxiety levels and sleep quality. Through this study, we aspire to offer a pragmatic intervention strategy to address anxiety and sleep-related challenges in isolated work environments, thereby enhancing the mental well-being of medical staff.

## 2 Methods

### 2.1 Study design and participants

We conducted a pre-post self-controlled trial, which received approval from the Ethics Committee of Ya’an People’s Hospital. Participants, including doctors and nurses who assisted in a mobile cabin hospital in Xinjiang Uyghur Autonomous Region, China, in October 2022, were recruited. All participants provided written informed consent, considering that PMR was not anticipated to induce physical harm. Inclusion criteria consisted of individuals who: (1) held a valid medical practitioner or nurse qualification issued by the Chinese National Health Commission; (2) were aged between 18 and 50 years; (3) had a minimum of six months of professional work experience; (4) participated in the management of COVID-19 patients; (5) voluntarily enrolled in PMR and underwent a standardized assessment of anxiety and sleep quality. And exclusion criteria encompassed individuals with (1) a history of psychiatric disorders, particularly anxiety and sleep disorders; (2) neurological conditions such as brain trauma, epilepsy, or severe somatic diseases; (3) a history of dependence on psychoactive substances; (4) current pregnancy or lactation. During the study, two independent researchers conducted in-person interviews using standardized questionnaires to collect demographic data and assess anxiety levels and sleep quality for each participant. Anxiety levels and sleep quality of participants were re-assessed on the 7th day after the intervention ended.

### 2.2 Questionnaire

#### 2.2.1 General demographic questionnaire

For the acquisition of demographic information, a general demographic questionnaire was formulated after reviewing relevant literature. The questionnaire encompassed terms including sex, age, prior occupational roles, current position within the cabin, years of education, marital status, history of smoking, history of alcohol consumption, and regular exercise (defined as involvement in physical activities at least three times a week, with each session lasting no less than 30 min).

#### 2.2.2 The Pittsburgh sleep quality index (PSQI)

PSQI (Buysse et al., 1989) is a standardized questionnaire consisting of 19 self-reporting items designed to assess seven components of sleep quality experienced over the past month. These components are categorized as follows: A. Subjective sleep quality; B. Sleep latency (the time taken to fall asleep); C. Sleep duration; D. Habitual sleep efficiency (the ratio of time asleep to time spent in bed); E. Sleep disturbances; F. Sleep medicine use; and G. Daytime dysfunction. To complete the PSQI, participants rated their sleep quality and experiences using a Likert scale, with response options ranging from 0 to 3. The total score of PSQI spans from 0 to 21, with higher scores indicative of poorer sleep quality. A total score ≤ 5 is considered indicative of normal sleep quality, while scores exceeding this threshold suggest poor sleep (Backhaus et al., 2002). For descriptive purposes, the total scores of 6–10, 11–15, and ≥ 16, were defined as mild, moderate, and severe poor sleep, respectively. A well-validated Chinese version of PSQI was employed, which exhibited a Cronbach’s α of 0.71 and an intraclass correlation coefficient of 0.90 (Ho et al., 2021).

#### 2.2.3 The Hamilton anxiety rating scale (HAMA)

HAMA (Hamilton, 1959) consists of 14 items that cover a broad spectrum of anxiety symptoms. The items assess various symptoms, including: (1) anxious mood, (2) tension, (3) fears, (4) insomnia, (5) cognitive changes, (6) depression, (7) somatic muscular and (8) sensory complaints, (9) cardiovascular, (10) respiratory, (11) gastrointestinal, (12) genitourinary, and (13) autonomic symptoms, along with (14) behavior during the interview. Among these, items (1) ~ (6) and (14) are categorized as psychological anxiety manifestations, while items (7) ~ (13) are classified as somatic manifestations. Participants rated each item on a severity scale ranging from 0 to 4. The total score of HAMA ranges from 0 to 56. HAMA total scores within the ranges of 8–17, 18–24, and ≥ 25 are classified as mild, moderate, and severe anxiety, respectively, Scores ≤7 are considered indicative of normal anxiety levels (Temiz et al., 2021). The Chinese version of HAMA utilized in this study has demonstrated good reliability and validity in previous research (Zhou et al., 2020).

The PSQI and HAMA assessments required approximately 15–20 min for completion and were administered in a paper-and-pencil format. For this study, both the PSQI and HAMA were modified to assess sleep and anxiety conditions over the preceding week.

### 2.3 Intervention

The intervention started on the second day following the baseline assessment. All participants engaged in daily PMR sessions for seven consecutive days before bedtime. The duration of a PMR session ranged from 25 to 30 min. Two researchers underwent standardized training for 3 days to thoroughly grasp each step of PMR (Liu et al., 2020; Xiao et al., 2020) to effectively guide each participant through the procedure. The PMR involved the following six steps in detail, guided by verbal instructions and simultaneous body movements from the two researchers:

1. Preparation: Participants, having changed into loose and comfortable attire, were escorted by the research personnel to a serene and comfortable, disturbance-free resting room where they assumed a comfortable seated position. The room temperature was regulated to 25°C using air conditioning.
2. Identification of Muscle Groups: The researchers guided participants in recognizing the primary muscle groups in their bodies (including the forehead, jaw, neck and shoulders, arms and hands, chest and abdomen, buttocks, and legs and feet) that could be progressively tensed and then relaxed. It is noteworthy that despite the participants in this study being healthcare professionals with anatomical knowledge, guidance from research personnel was still necessary to ensure the homogeneity of the intervention method.
3. Tension phase: Under the guidance of the researchers, participants initiated the process by focusing on the muscles in their forehead. Deliberately tensing this muscle group as much as they comfortably could, they kept the rest of their body relaxed. The tension was sustained for approximately 5 to 10 s, with participants concentrating on the sensations of tension within that specific muscle group.
4. Relaxation phase: After the tension phase, participants released the tension in the targeted muscle group suddenly and completely. They then directed their focus to the sensations of relaxation as the muscle group became loose and relaxed. This relaxed state was maintained for approximately 10 to 15 s, with participants concentrating on the feeling of relaxation within that specific muscle group.
5. Progression: Participants systematically moved on to the next muscle group, adhering to the same pattern of tensing and relaxing. They gradually worked through each muscle group, transitioning from one part of the body to another, such as the jaw, neck and shoulders, arms and hands, etc. A relaxed state was maintained between each muscle group.
6. Full-body relaxation: Once participants had progressed through all the major muscle groups, they allocated a few minutes to focus on their entire body. Imagining a wave of relaxation washing over their body, from the top of their head to the tips of their toes, participants breathed deeply and slowly, allowing themselves to sink into a profound state of relaxation.

### 2.4 Statistical analysis

For non-normally distributed variables, statistical descriptions were presented using the median and interquartile [*M* (*P*_25_, *P*_75_)]. The samples were grouped according to baseline data (i.e., several potential risk factors previously described), and inter-group comparisons of HAMA and PSQI were conducted using the Mann–Whitney U test or Kruskal-Wallis H test, as appropriate. As these included factors did not show statistical significance, further multivariate analysis or stratified analysis was not conducted. Pre-and post-intervention paired-design comparisons of HAMA and PSQI were conducted using the Wilcoxon test. Spearman’s rank correlation coefficient was employed for correlation analysis. Categorical data and ranked data were presented as *n* (%). Intergroup comparisons of ranked data were performed using the Wilcoxon test. All statistical tests were two-tailed, with a significance level set at *α* = 0.05 (*p* < 0.05 indicating statistical significance). IBM SPSS 24.0 software was utilized for data analysis, and GraphPad Prism 8.0.1 was employed for data visualization.

## 3 Results

### 3.1 Baseline PSQI and HAMA of participants with different demographic characteristics

The study period spanned from October 1, 2022, to October 14, 2022. 101 participants meeting the inclusion criteria were recruited in total. However, seven participants withdrew from the study due to job reassignment, culminating in a final sample size of 94. Consequently, the questionnaire completion rate was 93.1%. No significant differences were identified in PSQI or HAMA based on sex, age, educational years, or other general information (*p* > 0.05), as shown in Table 1.

**Table 1:** Comparison of PSQI and HAMA total scores at baseline (*n* = 94).

Variables		*n* (%)	PSQI			HAMA		
			Total score	*Z* / *χ*^2^	*p*	Total score	*Z* / *χ*^2^	*p*
Sex				−1.169	0.242		−0.287	0.774
	Male	20 (21.3)	10.0 (9.0, 12.0)			8.5 (4.0, 16.0)		
	Female	74 (78.7)	11.0 (7.0, 13.0)			8.0 (5.0, 12.0)		
Age				−1.307	0.191		−1.009	0.313
	18 ~ 30 y	46 (48.9)	11.0 (8.0, 13.0)			8.0 (6.0, 16.0)		
	31 ~ 50 y	48 (51.1)	9.0 (7.5, 12.0)			8.0 (5.0, 11.5)		
Prior occupation				−0.363	0.716		−0.806	0.420
	Doctor	22 (23.4)	9.0 (8.0, 12.0)			7.5 (4.0, 13.0)		
	Nurse	72 (76.6)	10.5 (7.0, 13.0)			8.5 (5.0, 12.5)		
Position in cabin				3.459	0.177		1.209	0.546
	Administrator	26 (27.7)	11.5 (9.0, 14.0)			9.0 (5.0, 16.0)		
	Doctor	18 (19.1)	9.0 (8.0, 11.0)			8.0 (5.0, 13.0)		
	Nurse	50 (53.2)	10.0 (7.0, 12.0)			8.0 (5.0, 11.0)		
Educational years				−0.222	0.824		−0.453	0.650
	14 ~ 15 y	19 (20.2)	10.0 (8.0, 13.0)			8.0 (5.0, 10.0)		
	≥16 y	75 (79.8)	10.0 (8.0, 12.0)			8.0 (5.0, 13.0)		
Marital status				−0.187	0.851		−0.773	0.439
	Married	77 (81.9)	10.0 (8.0, 13.0)			8.0 (5.0, 13.0)		
	Unmarried	17 (18.1)	11.0 (8.0, 12.0)			8.0 (5.0, 9.0)		
Smoking history				−0.501	0.617		−0.227	0.820
	Yes	12 (12.8)	9.0 (8.5, 13.5)			8.0 (3.5, 16.5)		
	No	82 (87.2)	10.0 (7.0, 12.0)			8.0 (5.0, 12.0)		
Alcohol consumption				−0.248	0.804		−0.423	0.672
	Yes	27 (28.7)	9.0 (8.0, 12.0)			8.0 (6.0, 14.0)		
	No	67 (71.3)	10.0 (8.0, 13.0)			8.0 (5.0, 12.5)		
Regular exercise				−1.797	0.072		−1.704	0.088
	Yes	29 (30.9)	9.0 (6.0, 12.0)			7.0 (4.0, 12.0)		
	No	65 (69.1)	10.0 (8.0, 13.0)			9.0 (6.0, 14.0)		

The total scores of PSQI and HAMA are both described as the median (P_25_, P_75_). PSQI, The Pittsburgh sleep quality index; HAMA, the Hamilton anxiety rating scale.

### 3.2 Correlation between PSQI and HAMA at baseline

Spearman’s rank correlation analysis revealed a significant correlation between the sleep quality and anxiety levels of participants at baseline. The total score of the PSQI and its components (except for dimension F: sleep medication) exhibited a positive correlation with the total score of the HAMA and its psychological anxiety component (*p* < 0.01). Additionally, a correlation was also observed between somatic anxiety manifestations and several components of the PSQI (see Table 2).

**Table 2:** Spearman rank correlation analysis between PSQI and HAMA at baseline (*n* = 94).

	PSQI total	A	B	C	D	E	F	G
HAMA total	0.518^**^	0.414^**^	0.351^**^	0.324^**^	0.354^**^	0.474^**^	0.089	0.364^**^
H	0.564^**^	0.435^**^	0.355^**^	0.398^**^	0.416^**^	0.462^**^	0.105	0.414^**^
I	0.255^*^	0.259^*^	0.209^*^	0.130	0.153	0.309^**^	0.036	0.118

All data represent Spearman rank correlation coefficients (r_s_). ^*^p < 0.05, ^**^p < 0.01. PSQI, The Pittsburgh sleep quality index; A. Subjective sleep quality; B. Sleep latency (the time taken to fall asleep); C. Sleep duration; D. Habitual sleep efficiency (the ratio of time asleep to time spent in bed); E. Sleep disturbances; F. Use of sleep medication; and G. Daytime dysfunction; HAMA, the Hamilton anxiety rating scale; H. psychological anxiety manifestations; I. somatic manifestations.

### 3.3 Comparison of sleep quality and anxiety pre- and post-intervention

**Figure 1:**
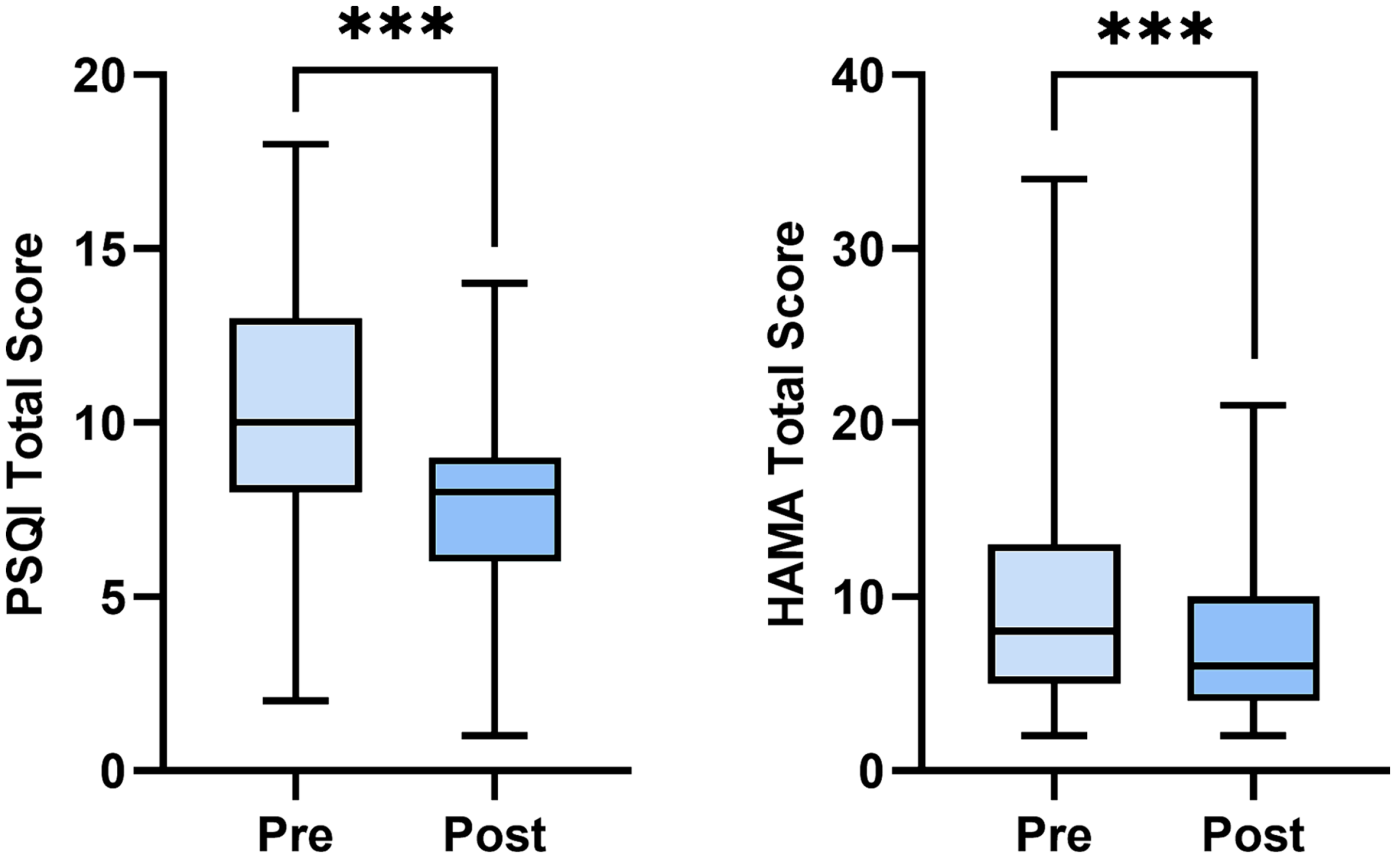
Changes of Pittsburgh sleep quality index (PSQI) and Hamilton anxiety scale (HAMA) score pre and post intervention. PSQI, The Pittsburgh sleep quality index; HAMA, The Hamilton anxiety rating scale. *n* = 94. ***p < 0.001.

**Figure 2:**
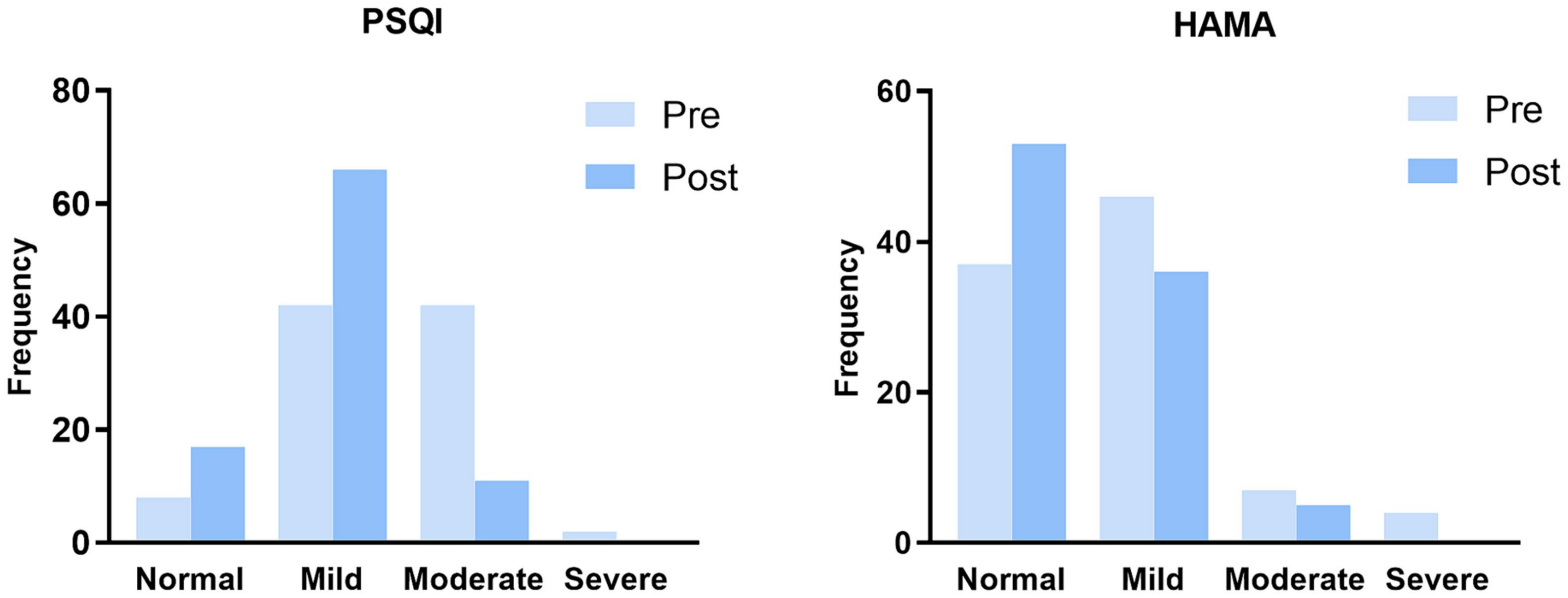
Frequency of sleep quality and anxiety pre and post intervention. PSQI, The Pittsburgh sleep quality index; HAMA, The Hamilton anxiety rating scale. *n* = 94.

The scores of both the PSQI total and the HAMA total decreased significantly before and after the intervention (both *p* < 0.001), as shown in Table 3 and Figure 1. After the intervention, the detection rates of poor sleep and anxiety symptoms, as well as the severity of the conditions, were significantly reduced among participants compared to pre-intervention (*p* < 0.001), as presented in Table 4 and Figure 2.

**Table 3:** Comparison of PSQI and HAMA scores pre and post intervention (*n* = 94).

	Before intervention	After intervention	*Z*	*p*
PSQI	10.0 (8.0, 13.0)	8.0 (6.0, 9.0)	−7.592	<0.001
HAMA	8.0 (5.0, 13.0)	6.0 (4.0, 9.5)	−6.199	<0.001

All data are median (P_25_, P_75_).

PSQI, The Pittsburgh sleep quality index; HAMA, the Hamilton anxiety rating scale.

**Table 4:** Detection rates of poor sleep and anxiety status pre and post intervention (*n* = 94).

		Before intervention	After intervention	*Z*	*P*
PSQI				−6.487	<0.001
	Normal	8 (8.5)	17 (18.1)		
	Mild	42 (44.7)	66 (70.2)		
	Moderate	42 (44.7)	11 (11.7)		
	Severe	2 (2.1)	0		
HAMA				−4.596	<0.001
	Normal	37 (39.4)	53 (56.4)		
	Mild	46 (48.9)	36 (38.3)		
	Moderate	7 (7.4)	5 (5.3)		
	Severe	4 (4.3)	0		

All data are *n* (%). PSQI, The Pittsburgh sleep quality index; HAMA, the Hamilton anxiety rating scale.

## 4 Discussion

This study aims to investigate the anxiety levels and sleep quality of healthcare practitioners involved in the management of COVID-19 patients in a mobile cabin hospital and to explore the impact of PMR on their anxiety levels and sleep quality. The results indicated that sleep and anxiety issues were relatively common among healthcare professionals in mobile cabin hospitals, and PMR was proven to be an effective intervention for improving sleep quality and alleviating anxiety.

COVID-19 is a newly emerged acute respiratory infectious disease with high contagion. Therefore, in China, diagnosed patients need to undergo isolation treatment in mobile cabin hospitals during the outbreak of novel coronavirus pneumonia (Sun et al., 2023). Even after undergoing isolation management, patients might still harbor concerns, such as whether they can truly recover, the possibility of aftereffects, and the risk of recurrence or infecting others. Research has reported a significant increase in the risk of anxiety and sleep problems during the COVID-19 pandemic and interpersonal isolation. However, this increased risk is not limited to patients; it also extends to healthcare professionals providing essential medical services in mobile cabin hospitals (Wang et al., 2020). The anxiety levels and sleep quality of healthcare professionals working extensively in modular hospitals for numerous COVID-19 patients should also be a focal point of attention.

In our study, the pre-intervention questionnaire results revealed no statistically significant differences in sleep quality and anxiety levels among participants based on demographic characteristics (Table 1). However, a correlation was found between participants’ sleep quality and the psychological anxiety component of HAMA, along with a correlation between somatic anxiety manifestations and several components of the PSQI (Table 2). Additionally, the baseline detection rates for poor sleep quality and anxiety status were notably high, at 91.5% (86/94) and 60.6% (57/94), respectively (Table 4 and Figure 2). Çürük et al. (2023) reported that 51.8% of nursing students experienced COVID-19-associated sleep problems, and 70.9% had COVID-19-related anxiety (39.9% mild, 20.8% moderate, and 10.2% severe), with a significant positive correlation between the fear of COVID-19 and anxiety and sleep disturbances. Another study during the COVID-19 pandemic found prevalence rates of short sleep and insomnia among 813 healthcare workers to be 38.8 and 72.8%, respectively, with sleep quality being associated with acute stress, depression symptoms, and anxiety (Diaz et al., 2021). Healthcare professionals in field hospitals often face a high workload, long and irregular working hours, night shifts, lack of support networks, direct exposure to COVID-19 cases, and other job-related challenges, which may contribute to anxiety and poor sleep among healthcare professionals (Romiti et al., 2023). Besides, the high prevalence of sleep and mental symptoms may also be influenced by personal or habit changes (Proserpio et al., 2022).

Furthermore, our study revealed that after PMR intervention, participants showed a significant reduction (*p* < 0.001) in the total scores of PSQI and HAMA (Table 3; Figure 1), as well as the detection rates of sleep impairment and anxiety states categorized by severity (Table 4; Figure 2). This suggests that PMR assists healthcare professionals in improving sleep quality and ameliorating anxiety. To the best of our knowledge, our study represents the first investigation into the effects of PMR on sleep quality and anxiety levels among healthcare professionals in isolated environments. PMR can help balance the sympathetic nervous system by promoting relaxation of the body, stimulating blood circulation, and ensuring muscle relaxation (Ferendiuk et al., 2019; Seid et al., 2023). Ozgundondu and Gok Metin (2019) and Gallego-Gómez et al. (2020) reported the effectiveness of PMR in alleviating anxiety-related sleep disturbances in critical care nurses and nursing students, respectively, leading to improved academic performance. Existing research primarily focuses on using PMR to alleviate patient pain. Yoo et al. (2022) conducted a randomized controlled trial on 37 fibromyalgia syndrome patients (PMR group = 18, non-PMR group = 19) over 8 weeks, indicating that PMR effectively alleviates pain and reduces fatigue. Another trial on 104 cancer patients found that PMR combined with interactive guided imagery served as a complementary therapy with positive effects on cancer pain relief (De Paolis et al., 2019). These studies demonstrate that PMR not only improves complications by relieving pain, such as psychological distress like anxiety and depression but also benefits gastrointestinal and central nervous system signs (Izgu et al., 2020; Eymir et al., 2022). Similar to previous evidence studying other populations, our findings suggest that healthcare management departments should pay attention to the psychological state and sleep quality of healthcare professionals before they are exposed to isolated environments. For individuals experiencing anxiety or sleep issues, PMR can be used to address these issues.

Several limitations exist in this study. Firstly, our study did not investigate other psychological variables that could potentially influence the sleep quality and anxiety levels, alongside with the outcomes of interest and the efficacy of PMR, such as family environment, socioeconomic status, occupational demands, body fat percentage, and body mass index (Özlü et al., 2021; Wang et al., 2024). Further investigation of these variables is warranted to be to provide a more comprehensive understanding of the factors impacting healthcare practitioners’ sleep quality, anxiety levels, and PMR effectiveness. Secondly, although our results indicated the effectiveness of PMR in improving negative emotions and sleep quality among healthcare professionals, this study was conducted in a single mobile cabin hospital with a small sample size, which may not be generalizable to individuals in other work environments, necessitating further multicenter study. In addition, the impact of PMR on the sleep and anxiety of the study subjects may be short-term, and its benefits over time remain uncertain. Future research can extend the observation period and adjust and optimize the details of the PMR procedure, intervention time, and frequency based on actual conditions. Lastly, relying solely on PMR to address sleep and anxiety issues may be insufficient, and future research can explore the combined effects of various interventions (such as comprehensive pre-job training, reading, auditory stimulation via music, engagement in interactive gaming, etc.) in managing these issues (Feng et al., 2022).

In summary, our study indicates prominent sleep and anxiety issues among healthcare practitioners in mobile cabin hospitals. As a simple and cost-effective self-training technique, PMR significantly improves the anxiety and sleep quality of healthcare professionals during the support period in mobile cabin hospitals. However, trials with larger samples are necessitated to further affirm these preliminary findings.

## Data availability statement

The raw data supporting the conclusions of this article will be made available by the authors, without undue reservation.

## Ethics statement

The studies involving humans were approved by the Ethics Committee of Ya’an People’s Hospital. The studies were conducted in accordance with the local legislation and institutional requirements. The participants provided their written informed consent to participate in this study.

## Author contributions

YL: Writing – original draft. JD: Writing – original draft. JuW: Writing – original draft. PL: Writing – original draft. ZS: Writing – review & editing. YH: Writing – review & editing. GC: Writing – review & editing. KH: Writing – review & editing. JiW: Writing – review & editing.
